# StreptoBase: An Oral *Streptococcus mitis* Group Genomic Resource and Analysis Platform

**DOI:** 10.1371/journal.pone.0151908

**Published:** 2016-05-03

**Authors:** Wenning Zheng, Tze King Tan, Ian C. Paterson, Naresh V. R. Mutha, Cheuk Chuen Siow, Shi Yang Tan, Lesley A. Old, Nicholas S. Jakubovics, Siew Woh Choo

**Affiliations:** 1 Genome Informatics Research Laboratory, High Impact Research Building (HIR) Building, University of Malaya, 50603 Kuala Lumpur, Malaysia; 2 Department of Oral Biology and Biomedical Sciences, Faculty of Dentistry, University of Malaya, 50603 Kuala Lumpur, Malaysia; 3 Oral Cancer Research and Coordinating Centre, Faculty of Dentistry, University of Malaya, 50603 Kuala Lumpur, Malaysia; 4 Center for Oral Health Research, School of Dental Sciences, Newcastle University, Framlington Place, Newcastle upon Tyne, United Kingdom; 5 Genome Solutions Sdn Bhd, Suite 8, Innovation Incubator UM, Level 5, Research Management & Innovation Complex, University of Malaya, 50603 Kuala Lumpur, Malaysia; University of Kansas Medical Center, UNITED STATES

## Abstract

The oral streptococci are spherical Gram-positive bacteria categorized under the phylum *Firmicutes* which are among the most common causative agents of bacterial infective endocarditis (IE) and are also important agents in septicaemia in neutropenic patients. The *Streptococcus mitis* group is comprised of 13 species including some of the most common human oral colonizers such as *S*. *mitis*, *S*. *oralis*, *S*. *sanguinis* and *S*. *gordonii* as well as species such as *S*. *tigurinus*, *S*. *oligofermentans* and *S*. *australis* that have only recently been classified and are poorly understood at present. We present StreptoBase, which provides a specialized free resource focusing on the genomic analyses of oral species from the mitis group. It currently hosts 104 *S*. *mitis* group genomes including 27 novel mitis group strains that we sequenced using the high throughput Illumina HiSeq technology platform, and provides a comprehensive set of genome sequences for analyses, particularly comparative analyses and visualization of both cross-species and cross-strain characteristics of *S*. *mitis* group bacteria. StreptoBase incorporates sophisticated in-house designed bioinformatics web tools such as Pairwise Genome Comparison (PGC) tool and Pathogenomic Profiling Tool (PathoProT), which facilitate comparative pathogenomics analysis of *Streptococcus* strains. Examples are provided to demonstrate how StreptoBase can be employed to compare genome structure of different *S*. *mitis* group bacteria and putative virulence genes profile across multiple streptococcal strains. In conclusion, StreptoBase offers access to a range of streptococci genomic resources as well as analysis tools and will be an invaluable platform to accelerate research in streptococci. **Database URL**: http://streptococcus.um.edu.my.

## Introduction

*Streptococcus* is a major genus of spherical Gram-positive bacteria which belong to the phylum *Firmicutes*. Streptococci are classified as alpha-hemolytic, beta-hemolytic or gamma-hemolytic according to their appearance on blood agar. Alpha-hemolysis involves the bleaching of heme iron by streptococcal hydrogen peroxide (H_2_O_2_), resulting in a greenish tinge on blood agar [1]. Alpha-hemolytic streptococci used to be known as the ‘Viridans group’ for the greenish color produced by hemolysis. However, alpha-hemolysis is not entirely consistent between different strains of individual Streptococcal species, and therefore the term ‘Viridans’ is somewhat misleading and is no longer used. These organisms are now more commonly known as the oral streptococci. Overall, the streptococci are divided into six groups, namely the Mitis, Anginosus, Salivarius, Mutans, Bovis and Pyogenic groups, using sequence analysis of the 16S rRNA gene or of a group of housekeeping genes [2–4]. In 2002, Facklam proposed a phenotypic identification scheme which included an additional new cluster called Sanguinis [5]. This cluster, containing *S*. *sanguinis*, *S*. *gordonii* and *S*. *sinensis* is sometimes included within the mitis group.

The human oral streptococci are commensals which often inhabit the gastrointestinal and genitourinary tracts, as well as the oral mucosa and tooth surfaces. In healthy individuals, streptococci can constitute more than 50% of the oral microbiota [6] and these bacteria generally possess low pathogenic potential. However, oral streptococci can invade the bloodstream, and have the potential to cause infective endocarditis (IE) or post-antineoplastic septicaemia in neutropenic patients with haematological disease. Other oral *Streptococcus*-associated conditions including odontofacial infections, brain abscesses and abdominal infections have also been reported [7]. Furthermore, recent work has shown that *S*. *mitis* group bacteria play a major role in exacerbating influenza infection particularly among immunocompromised individuals; *Streptococcus oralis* and *S*. *mitis* were found to produce neuraminidase (NA), a vital target of anti-influenza drugs. The NA activity exhibited by these oral bacteria stimulates the release of influenza virus, boosts viral M1 protein expression levels and activates the cell signaling ERK pathway, potentially enhancing viral infections [8].

The mitis group is comprised of 13 known species including *S*. *australis*, *S*. *cristatus* (formerly *S*. *crista*), *S*. *gordonii*, *S*. *infantis*, *S*. *mitis*, *S*. *oligofermentans*, *S*. *oralis*, *S*. *parasanguinis* (formerly *S*. *parasanguis*), *S*. *peroris*, *S*. *pneumoniae*, *S*. *pseudopneumoniae*, *S*. *sanguinis* (formerly *S*. *sanguis*), and the latest grouped species, *S*. *tigurinus*. Currently, the complete genome sequences of 7 species of this mitis group (*S*. *pneumoniae*, *S*. *pseudopneumoniae*, *S*. *mitis*, *S*. *oralis*, *S*. *gordonii*, *S*. *sanguinis* and *S*. *parasanguinis*) are stored on the National Center for Biotechnology Information (NCBI)’s FTP site.

Here, we present StreptoBase which provides an invaluable resources and analysis platform for research communities. Through this platform and the provided in-house designed analysis tools, users may obtain insights into the biology, phylogeny, genetic variation and virulence of particular strains or species of interest. Furthermore, we have included 27 newly sequenced, assembled and annotated genomes of novel strains from six different species of *S*. *mitis* group from our laboratory into StreptoBase. These new genomes include novel genome sequences of the recently classified species *S*. *oligofermentans* and *S*. *tigurinus*. The ultimate objective of StreptoBase is to provide a user-friendly database resource and analysis platform. Users can search, browse, visualize, download and analyze the mitis group genomes, particularly comparative whole-genome analysis on the fly using our in-house advanced bioinformatics tools, which is designed to support the expanding *Streptococcus* genus research community.

## Materials and Methods

### Datasets

Seventy-seven genome sequences of *S*. *mitis* group bacteria were downloaded from the public NCBI database. We also have included 27 novel strains/genomes of *S*. *mitis* group generated from our laboratory in a sequencing project. All 27 strains were clinical isolates from individuals with dental plaque or infective endocarditis from different geographical locations (Table 1). Of these strains, 14 strains were isolated in the United Kingdom, 10 in United States, 2 in Australia and 1 in Denmark (Table 1). *S*. *sanguinis* NCTC 7863 is also known as ATCC 10556 while *S*. *gordonii* Blackburn and Channon are designated NCTC 10231 and NCTC 7869, respectively. Additionally, a number of these *S*. *mitis* group strains including JPIIBBV4, JPIIBV3, JPIBVI, LRIIBV4, DGIIBVI and DOBICBV2 have been previously described [9]. The isolation of strain M99 was described in a study of mechanisms of platelet aggregation by oral streptococci [10]. The other two oral isolates, SK120 and SK184 have also been described by Mogens Kilian and his fellow researchers in their taxonomic study of ‘Viridans’ Streptococci conducted in 1989 [11].

**Table 1:** The isolation details of 27 *Streptococcus* strains includes isolation source, geographical area and strain author.

Strain Name	Identified Species	Isolation source	Country	Strain Author	References
**PV40**	*S*. *gordonii*	Infective endocarditis	UK	P.M. Vesey, S.D. Hogg and R.R.B. Russell, Newcastle University	
**NCTC 7863**	*S*. *sanguinis*	Infective endocarditis	USA	White and Niven 1946	Streptococcus sanguinis (ATCC^®^ 10556^™^)
**Blackburn**	*S*. *gordonii*	Human isolate	UK	R. Hare, P.H.L.S. Colindale, London	Describe in Nobbs, A. H., et al (2007). Journal of bacteriology, 189(8), 3106–3114.
**BVME8**	*S*. *parasanguinis*	Human oral cavity	UK	J. Manning, S.D. Hogg, Newcastle University	
**Channon**	*S*. *gordonii*	Not recorded	UK	R. Hare, Queen Charlotte’s Hospital, London	Described in Millsap, K. W. et al (1999). FEMS Immunology & Medical Microbiology, 26(1), 69–74.
**DGIIBVI**	*S*. *tigurinus*	Dental plaque	USA	M. Levine, Oklahoma University	Described in McAnally & Levine (1993) Oral Microbiol Immunol 8: 69–74
**DOBICBV2**	*S*. *oligofermentans*	Dental plaque	USA	M. Levine, Oklahoma University	Described in McAnally & Levine (1993) Oral Microbiol Immunol 8: 69–74
**FSS2**	*S*. *gordonii*	Infective endocarditis	UK	S.D. Hogg, Newcastle University	
**FSS3**	*S*. *gordonii*	Infective endocarditis	UK	S.D. Hogg, Newcastle University	
**FSS4**	*S*. *sanguinis*	Infective endocarditis	UK	S.D. Hogg, Newcastle University	
**FSS8**	*S*. *gordonii*	Infective endocarditis	UK	S.D. Hogg, Newcastle University	
**FSS9**	*S*. *sanguinis*	Infective endocarditis	UK	S.D. Hogg, Newcastle University	
**JPIIBBV4**	*S*. *oligofermentans*	Dental plaque	USA	M. Levine, Oklahoma University	Described in McAnally & Levine (1993) Oral Microbiol Immunol 8: 69–74
**JPIIBV3**	*S*. *oralis*	Dental plaque	USA	M. Levine, Oklahoma University	Described in McAnally & Levine (1993) Oral Microbiol Immunol 8: 69–75
**JPIBVI**	*S*. *tigurinus*	Dental plaque	USA	M. Levine, Oklahoma University	Described in McAnally & Levine (1993) Oral Microbiol Immunol 8: 69–76
**LRIIBV4**	*S*. *oligofermentans*	Dental plaque	USA	M. Levine, Oklahoma University	Described in McAnally & Levine (1993) Oral Microbiol Immunol 8: 69–77
**M5**	*S*. *gordonii*	Dental plaque	USA	Rosan, B., University of Pennsylvania	Described in Rosan B (1973) Infect Immun 7 (2):205
**M99**	*S*. *gordonii*	Infective endocarditis	USA	P.M. Sullam, UCSF	Isolation described in Sullam, P.M., Valone, F.H., and Mills, J. (1987) Infect Immun 55: 1743–1750.
**MB451**	*S*. *sanguinis*	Infective endocarditis	UK	S.D. Hogg, Newcastle University	
**MB666**	*S*. *gordonii*	Infective endocarditis	UK	S.D. Hogg, Newcastle University	
**MW10**	*S*. *gordonii*	Not recorded	Australia	J. Manning, Sydney Dental School	
**PJM8**	*S*. *sanguinis*	Human oral cavity	UK	J. Manning, S.D. Hogg, Newcastle University	
**PK488**	*S*. *gordonii*	Subgingival dental plaque	USA	P. E. Kolenbrander, National Institutes of Health, MD	
**POW10**	*S*. *parasanguinis*	Not recorded	Australia	J. Manning, Sydney Dental School	
**SK12**	*S*. *gordonii*	Human oral cavity	Denmark	M. Kilian, Aarhus, Denmark	
**SK120**	*S*. *gordonii*	Human oral cavity	UK	P. H. A. Sneath (provided by M. Kilian)	Described in Kilian et al (1989) INTERNATIONAL JOURNAL OF SYSTEMATIC BACTERIOLOGY, 39: 471–484.
**SK184**	*S*. *gordonii*	Dental plaque	UK	P. Handley (provided by M. Kilian)	Described in Kilian et al (1989) INTERNATIONAL JOURNAL OF SYSTEMATIC BACTERIOLOGY, 39: 471–484.

Briefly, the 27 *S*. *mitis* group genomes were sequenced using Next-Generation Sequencing Illumina HiSeq2000 platform. Data pre-processing was performed by a trimming approach (Phred score Q20) and assembled using CLC Genomic Workbench V6.5 (CLC BIO Inc., Aarhus, Denmark). In general, these assemblies showed high N50 values and low contig numbers, indicating high quality genome assemblies. The assembled mitis group genomes harbor an average GC content of 35% to 45% and with an average genome size of approximately 2MB (Table 2).

**Table 2:** The genome identity of the 27 isolated *Streptococcus* strains with the summary assembly results.

Strain Name	K-mer	Contig no.	N50 (bp)	Genome Size (bp)	Identified Species	Genome coverage (%)	Genome Identity (%)	NCBI Accession numbers
**PV 40**	32	43	233745	2191051	*S*. *gordonii*	95	98	SAMN03480623
**NCTC 7863**	24	110	45631	3078022	*S*. *sanguinis*	84	95	SAMN03480625
**Blackburn**	24	50	158790	2164532	*S*. *gordonii*	90	96	SAMN03480626
**BVME8**	17	109	53977	2122687	*S*. *parasanguinis*	86	97	SAMN03480630
**Channon**	28	33	174000	2233600	*S*. *gordonii*	89	96	SAMN03480628
**DGIIBVI**	26	44	229281	1885841	*S*. *tigurinus*	79	94	SAMN03480631
**DOBICBV2**	21	99	45179	1979216	*S*. *oligofermentans*	77	94	SAMN03480632
**FSS2**	28	19	575926	2185874	*S*. *gordonii*	92	98	SAMN03481559
**FSS3**	21	398	172943	2312061	*S*. *gordonii*	92	96	SAMN03481560
**FSS4**	28	63	389092	2312671	*S*. *sanguinis*	85	95	SAMN03480635
**FSS8**	25	41	286373	2151860	*S*. *gordonii*	90	95	SAMN03480641
**FSS9**	25	20	356680	2429261	*S*. *sanguinis*	97	95	SAMN03480643
**JPIIBBV4**	30	95	48467	1991853	*S*. *oligofermentans*	78	94	SAMN03480680
**JPIIBV3**	31	75	209178	1990145	*S*. *oralis*	79	94	SAMN03480681
**JPIBVI**	28	37	940267	1792994	*S*. *tigurinus*	87	96	SAMN03480682
**LRIIBV4**	24	373	44211	2097683	*S*. *oligofermentans*	76	94	SAMN03481561
**M5**	28	67	145888	2157832	*S*. *gordonii*	88	95	SAMN03480683
**M99**	29	45	134448	2167061	*S*. *gordonii*	89	95	SAMN03480687
**MB451**	26	27	382788	2452806	*S*. *sanguinis*	94	96	SAMN03480686
**MB666**	25	20	313888	2308142	*S*. *gordonii*	90	96	SAMN03480688
**MW10**	28	27	247835	2186113	*S*. *gordonii*	92	98	SAMN03480689
**PJM8**	25	163	396031	2368281	*S*. *sanguinis*	92	95	SAMN03480699
**PK488**	38	46	183297	2262708	*S*. *gordonii*	91	96	SAMN03480700
**POW10**	14	117	30074	2042518	*S*. *parasanguinis*	77	96	SAMN03480701
**SK12**	25	28	235294	2164760	*S*. *gordonii*	89	95	SAMN03480703
**SK120**	36	27	200167	2145851	*S*. *gordonii*	90	96	SAMN03480740
**SK184**	26	53	210865	2255121	*S*. *gordonii*	92	97	SAMN03480741

### Genome annotation

StreptoBase currently comprises a total of 104 *S*. *mitis* group genomes (a genome collection of NCBI resources genome records plus our 27 isolated strains) from 11 species: *S*. *australis*, *S*. *cristatus*, *S*. *gordonii*, *S*. *infantis*, *S*. *mitis*, *S*. *oligofermentans*, *S*. *oralis*, *S*. *parasanguinis*, *S*. *peroris*, *S*. *sanguinis*, and *S*. *tigurinus* (Table 3).

**Table 3:** The species table summarizes the total number of draft and complete genomes of each *S*. *mitis* group species accordingly.

Species	Draft Genomes	Complete Genome
*S*. *australis*	1	0
*S*. *cristatus*	1	0
*S*. *gordonii*	14	1
*S*. *infantis*	6	0
*S*. *mitis*	21	1
*S*. *oligofermentans*	3	1
*S*. *oralis*	10	1
*S*. *parasanguinis*	8	2
*S*. *peroris*	1	0
*S*. *sanguinis*	26	1
*S*. *tigurinus*	6	0

To facilitate comparative analysis across different *S*. *mitis* group genomes, consistency in annotation is important. Therefore, we annotated all 104 genome sequences using the Rapid Annotation using Subsystem Technology (RAST) pipeline, which is a well-established and fully open web-based engine, supporting annotation of both complete and draft genomes[12]. The RAST pipeline enables genome identification of an array set of distinct genome components including protein-coding genes, ribosomal RNAs (rRNAs) and transfer RNAs (tRNAs), pseudogenes, gene function prediction. The RAST genome annotation works by mapping a set of genes to their corresponding subsystems as well as their metabolic reconstructions. Moreover, it predicts functional proteins assignment according to their relatedness in the subsystems of FIGfams database. Using the RAST pipeline, we predicted 213,268 Coding Sequences (CDSs), 5,140 RNAs and 4,542 tRNAs in all 104 genomes in the mitis group genomes.

To systematically predict subcellular localization of each RAST-predicted gene, we utilized the latest PSORTb subcellular localization tool (version 3.0) program [13]. PSORTb is an efficient, open-source tool which supports high precision of proteome-scale prediction coverage and refined sub-categories localization. The predicted subcellular localization sites were computationally calculated based on the values of feature variables which infer the sequences characteristics. Each of the generated values was then sorted to their respective candidate site through their estimated relativity. Besides the subcellular localization information, we also ran our in-house Perl script to estimate the GC content, hydrophobicity and molecular weight of each protein or gene.

### Database structure, composition and implementation

**Fig 1:**
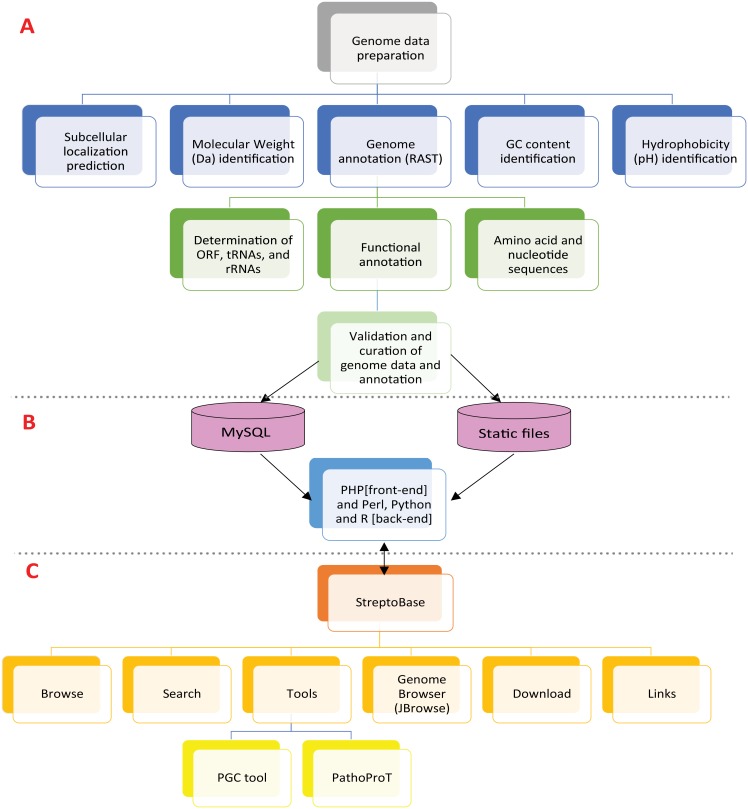
StreptoBase structure and composition. (A) Flowchart of functional annotation of *Streptococcus* genomes. (B) Diagram of the StreptoBase web server. (C) Web interface of the StreptoBase sitemap.

StreptoBase was designed to provide a wide range of useful information and functionalities (Fig 1). For instance, StreptoBase provides users with some background information about *S*. *mitis* group species. Within the homepage of StreptoBase, there is a summary box which comprises the genome information stored in the database, such as number of species, strains, number of CDS, number of RNAs and number of tRNAs (Table 4), which are useful before users proceed to further genome details and downstream analyses.

**Table 4:** StreptoBase Data Summary Table.

Database Summary	Counts
Number of Species:	11
Number of Strains/Genomes:	104
Number of CDS:	213,268
Number of RNAs:	5,140
Number of tRNAs:	4,542

Furthermore, we have compiled and gathered information from various sources on *S*. *mitis* group species, for example, news and conferences, blogs and information and recently published papers, which are available in the StreptoBase homepage. By clicking on “Browse” menu, users will see the list of 11 *S*. *mitis* group species along with their respective number of draft and complete genomes, while each “View Strains” button, enabling users to visualize all available *Streptococcus* genomes of any particular species, respectively. Under the “Browse Strains” page, a summarized genome description which encompasses genome size (Mbp), GC content (%) and a list of contigs, genes and rRNAs of that particular species strain are tabulated and displayed. The “Details” button allows users to access further detailed and additional data of that particular strain such as a complete list of ORFs in the genome, their corresponding functions, start and stop chromosomal positions of each ORF/gene in the “Browse ORF” page. To display all information about an ORF or gene, users can click on the “Details” button associated with the ORF. This will open the “ORF Detail” page, displaying information such as their gene type, start and stop positions, nucleotide length, amino acid sequences, functional classification, SEED subsystem (if available), direction of transcription (strand), subcellular localization, hydrophobicity (pH) as well as molecular weight (Da) will be displayed.

#### *Streptococcus* Genome Browser (SGB)

StreptoBase is equipped with a real-time and interactive *Streptococcus* Genome Browser (SGB), which was customised from a well-established genome browser, JBrowse [14], a fast and modern JavaScript-based genome browser which performs navigation on genome annotations and visualization of the location of genes and flanking genomic regions/genes of a selected *Streptococcus* strain. This interactive SGB enables users to browse genes or genomic regions with graphic-wise motion smoothly and rapidly. SGB overcomes the discontinuous transitions and provides efficient panning and zooming of a specific genomic region in each *Streptococcus* genome. Furthermore, users can remotely turn on or off the DNA, RNA, and CDS tracks during the navigation process, providing flexibility in customizing what to view in the SGB viewer. We have also implemented a “Search” feature in the genome browser page, allowing users to quickly search a gene by keyword or ORF ID which is not provided by JBrowse.

#### Real-time keyword search engine

Considering the fact that StreptoBase would host an extensive number of genes and their annotation and this information will increase periodically, the ability to rapidly search a gene in the database is crucial. To address this issue, we implemented a real-time search engine in StreptoBase using AJAX technology. This real-time search engine was designed to support asynchronous communications between web interface and MySQL database, avoiding the need to refresh the web page and allowing the visualization of search results seamlessly. The real-time search engine retrieves a list of suggested functional classifications of genes that are related to the entered keyword once a keyword is typed.

#### Database implementation

The web interface of StreptoBase was developed using HyperText Markup Language (HTML), HyperText Preprocessor (PHP), JavaScript, jQuery, Cascading Style Sheets (CSS) and AJAX. The StreptoBase is supported by Linux, Apache, MySQL and PHP (LAMP) architecture.

The Apache web server is equipped with Linux OS to manage the comprehensive *Streptococcus* genomic data housed in StreptoBase. The front end PHP framework of CodeIgniter version 2.1.3 was implemented to offer model-view-controller (MVC), dividing application data, presentation and background logic and process into three distinct modules. With this advanced feature, all *Streptococcus* related sources codes and biological data are arranged in a clear and organized fashion which facilitate future updating of new *Streptococcus* genomes into the existing database system. For *Streptococcus* biological data storage and management, we utilized MySQL version 14.12 in order to store the extensive *Streptococcus* genome information into a well-designed database schema and tables. The backend process of StreptoBase is monitored by Perl script, Python script and R script which support the efficiency and functionality of our integrated bioinformatics tools.

Additionally, users are able to download all the *Streptococcus* genome sequences, ORF annotation details in table format, ORF sequences, RNAs and CDSs as well as nucleotide and amino acid sequences via the “Download” menu.

## Results

### Database features and incorporated bioinformatics tools

The *S*. *mitis* group species are important colonizers of the oral cavity, and are occasionally associated with serious infections [15]. In addition, these organisms have recently been suggested to play important roles in the pathogenesis of influenza [8]. Therefore, the genomic study of diverse *S*. *mitis* group bacteria is essential in order to understand how these microorganisms transit from a commensal lifestyle in the mouth to subsequent pathogenesis. However, there is no existing specialized genome database available for the wide array of *S*. *mitis* group genomes for comparative genomics. While most biological genome databases only focus on the genome content and genetic variation, we have identified a need to create functional bioinformatics tools to investigate virulence determinants within genomes through comparative pathogenomics, as well as to compare the genome content and genetic variation within the *S*. *mitis* group bacteria.

#### Pairwise Genome Comparison (PGC) tool

We designed and customised a web-based PGC tool for *S*. *mitis* group bacteria, enabling users to select and perform pairwise comparisons between two user-selected *Streptococcus* genomes. A list of *Streptococcus* genomes is available on PGC tool of StreptoBase, allowing users to choose two *Streptococcus* genomes for cross strain or cross species comparison. Alternatively, users can upload their own genome sequences, either nucleotides or protein, and compare with the *Streptococcus* genomes in StreptoBase.

Briefly, the PGC pipeline is supported by NUCmer that is designed to align whole-genome sequences, and Circos that is a well-established tool for genome visualisation. Once users submit their jobs to our server, PGC will call NUCmer program to align user-selected genomes and in-house scripts will be used to process the genome alignment output and generate input files parsed to Circos in order to generate a circular ideogram layout of alignments. Unlike the conventional linear display of alignments, the circular layout shows the relationship between pairs of positions with karyotypes and links encoding the position, size and orientation of the related genomic elements.

Three user-defined parameters are provided in the PGC web interface including minimum percent identity (%), merge threshold (bp) and link threshold (bp). The minimum percent identity cut-off defines a homologous region (represented by links/ribbons in the Circos plot) between the two compared genomes. The merge threshold allows merging of two links/ribbons which have distance within the user-defined threshold, and the link threshold allows users to eliminate any mapped/homologous regions that have genomic size less than the user-defined cut-off. A histogram track is added in the outer ring of the circular plot to indicate the percentage of mapped regions, allowing users to quickly identify potential indels (indicated by white gaps) and mapping regions (indicated by green charts) between the two aligned genomes. The implementation of the PGC pipeline is governed using Perl scripts. This pipeline produces two types of outputs: NUCmer alignment results and the high quality Circos plot (SVG format). Users can freely download these results for publication or further analyses in the PGC result page.

The existing Microbial Genome Comparison (MGC) tool utilizes an in silico genome subtraction method to identify genetic elements specific to a group of strains [16]. While PGC tool uses genome files and NUCmer to perform pairwise genome alignment, the MGC tool uses in silico fragmented genome sequences and performs BLASTN on groups of queries. On the contrary, the VISTA Browser which is well-known for its biological application is able to perform pre-computed pairwise and multiple whole-genome alignments using both global and local alignments [17]. In contrast to circular plots and histograms that are generated by the PGC tool, the alignment results generated by VISTA Browser are displayed using VISTA track in graph plot format to show conserved regions. Additionally, the open source Java-based Artemis Comparison Tool (ACT) requires users to generate a comparison file which identifies homology regions between assembly and reference genome using programs such as BLASTN, TBLASTX or Mummer to be loaded on ACT [18]. The comparative ACT visualization is performed using Artemis components. By contrast, our PGC tool enables a single-flow process of pairwise genome alignment and instant display of the comparative alignment Circos plot.

**Fig 2:**
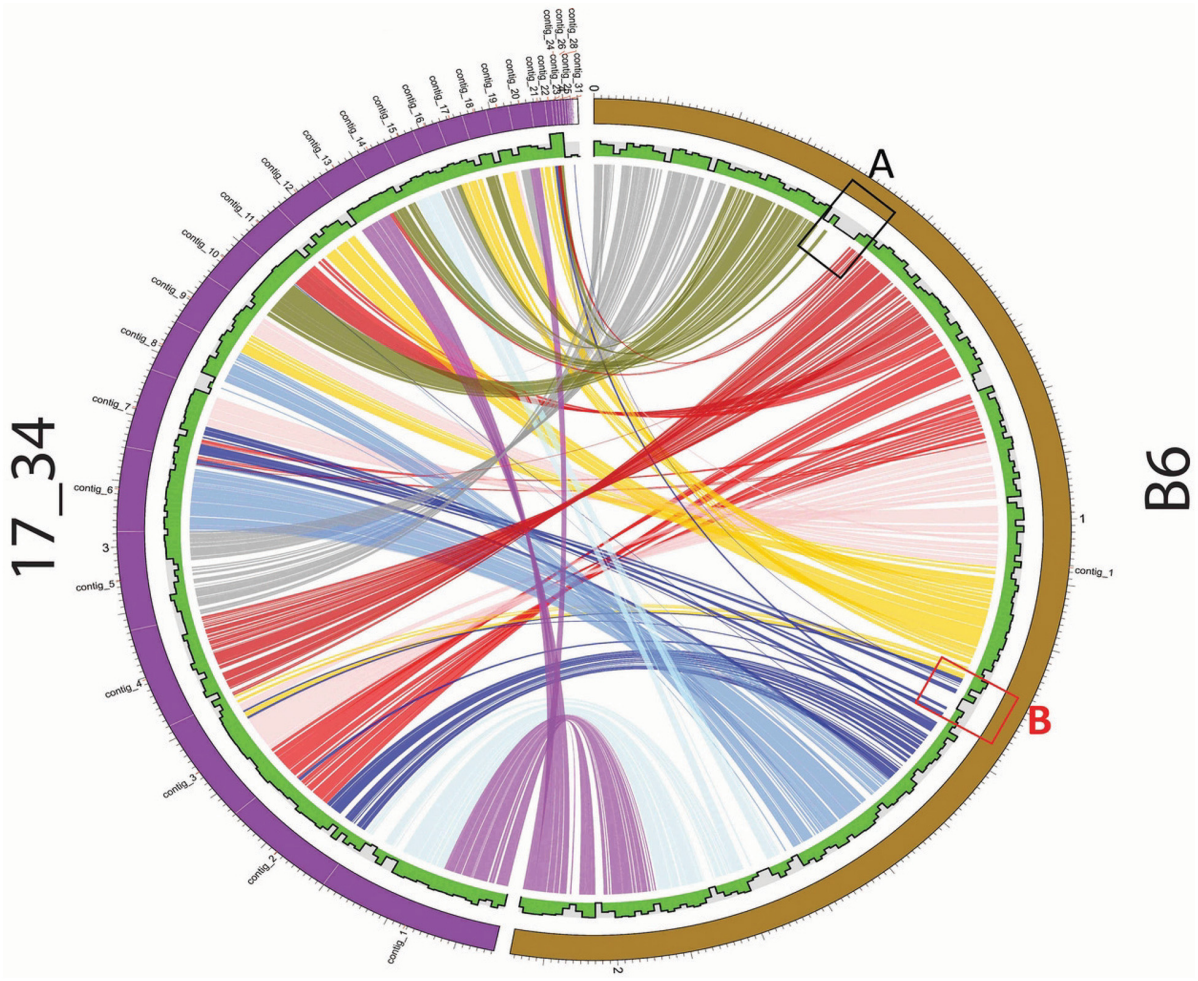
Pairwise genome comparison between *S*. *mitis* B6 *and S*. *mitis* 17/34 using PGC tool incorporated in StreptoBase. 50% sequence identity and 50% sequence coverage were used to compare strains using the PGC tool. A and B highlight the indels of the pairwise genome comparison between *S*. *mitis* B6 *and S*. *mitis* 17/34.

To demonstrate the utility of PGC, we compared *S*. *mitis* B6 (complete genome) and 17/34 (draft genome) as a case study in Fig 2.

The parameters were set as 80% of minimum percent, default value of 1000bp link threshold and 2000bp merge threshold. *S*. *mitis* B6 was isolated in Germany, whereas *S*. *mitis* 17/34 was isolated from the urethra of a Russian patient with urethritis. Based on the generated PGC plot, both *S*. *mitis* genomes generally shared high similarity as most of their genomic regions could be aligned (Fig 2). One of the features of PGC plot is its ability to quickly identify putative indels via visualization of the gaps in the plot chart which is supported by information displayed in the histogram track. For instance, two of the gap occurrences (Fig 2) indicate the absence of genomic regions in the *S*. *mitis* 17/34 genome. The external circular bar of the plot shows the genome size measurements which are approximately 2MB for both *S*. *mitis* genomes. Based on the gap observed in Fig 2 (indel ‘A’), the gene loss occurred close to position 400,000bp.

**Fig 3:**
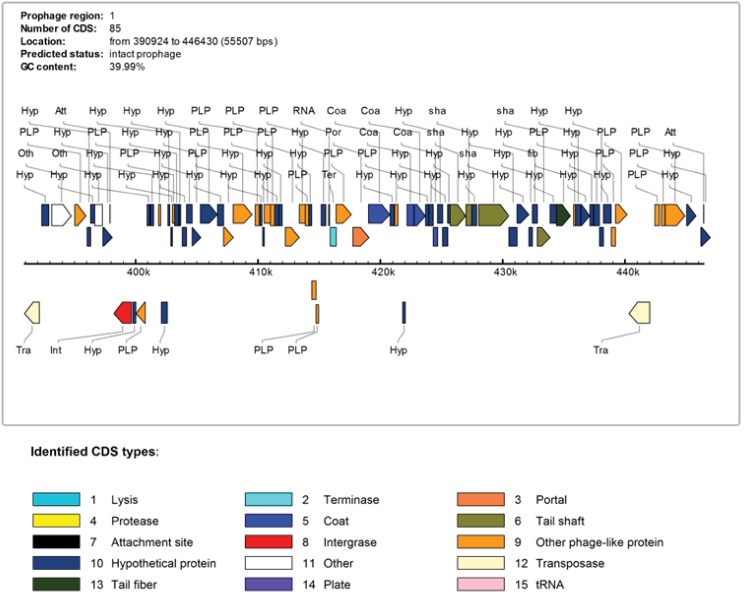
Intact prophage detected in *S*. *mitis* B6. This prophage has 85 predicted genes.

Next, we examined the genes located at indel ‘A’ in *S*. *mitis* B6 (Fig 2) by visualising this region using SGB. We identified many phage-related genes associated with this region. To further examine this region, we utilized PHAST (PHAge Search Tool) to annotate and identify prophages sequences found within *S*. *mitis* B6 genome (You Zhou et al., 2011). A 56Kb intact prophage with 82 CDSs and GC content of 39.9% was detected from 390,924bp to 446,969bp. Since *S*. *mitis* B6 is a complete genome, we can therefore imply the base pair position directly into our B6 annotation file. According to PHAST results, this intact prophage of *S*. *mitis* B6 comprised phage-associated genes including phage integrase protein, phage CI-like repressor, phage binding protein, phage portal protein, SPP1 family phage head morphogenesis protein and phage capsid proteins. Therefore, we suggest that *S*. *mitis* B6 might have recently acquired this intact prophage. The graphical display of the intact prophage with different types of phage-related genes is shown in Fig 3.

Based on the indel ‘B’ detected on the PGC plot in Fig 2, we have revealed a 24Kb incomplete prophage with GC content of 39.17% located at position 1356040bp to 1380128bp Interestingly, this region contains a complete *atp* operon regulated by the CcpA protein within this incomplete prophage of *S*. *mitis* B6 genome. The genes of the *atp* operon are shown in Table 5. These genes encoding ATP synthases are commonly possessed by oral streptococci for adaptation to the acidic host environment by creating a more alkaline internal system.

**Table 5:** The ATP synthases within the *atp* operon of *S*. *mitis* B6.

Locus Tag	Gene Name	Functional annotation
**smi_1315**	*atpE*	ATP synthase C chain (EC 3.6.3.14)
**smi_1314**	*atpB*	ATP synthase A chain (EC 3.6.3.14)
**smi_1313**	*atpF*	ATP synthase B chain (EC 3.6.3.14)
**smi_1312**	*atpH*	ATP synthase delta chain (EC 3.6.3.14)
**smi_1311**	*atpA*	ATP synthase alpha chain (EC 3.6.3.14)
**smi_1310**	*atpG*	ATP synthase gamma chain (EC 3.6.3.14)
**smi_1309**	*atpD*	ATP synthase beta chain (EC 3.6.3.14)
**smi_1308**	*atpC*	ATP synthase epsilon chain (EC 3.6.3.14)

This protective mechanism is critical especially for streptococcal acid-sensitive glycolytic enzymes [19]. Hence, it may be that the acquisition of this *atp* operon carried by the incomplete prophage of *S*. *mitis* B6 via horizontal gene transfer has assisted its commensal status in maintaining the optimal pH level for bioenergetics processes of *S*. *mitis* B6 cells.

#### Pathogenomics Profiling (PathoProT) tool

PathoProT was designed to predict virulence genes by comparing *Streptococcus* amino acid sequences against the Virulence Factors Database (VFDB) [20]. PathoProT utilizes the stand-alone BLAST tools downloaded from the NCBI website. VFDB (Version 2012) currently hosts a set of 19,775 experimentally verified virulence genes originating from a wide range of different bacterial species, providing a useful resource for sequence homology searches. Users can select a list of *Streptococcus* strains for comparative analysis and set the cut-off, for example, genome identity and completeness for the BLAST search through our provided online web form. The default parameters of PathoProT pipeline are set at 50% sequence identity and 50% sequence completeness for searching and identifying orthologous virulence genes across the selected *Streptococcus* genomes. However, users can apply their desired cut-offs for the homology search in order to achieve the optimal stringency levels in their analyses.

**Fig 4:**
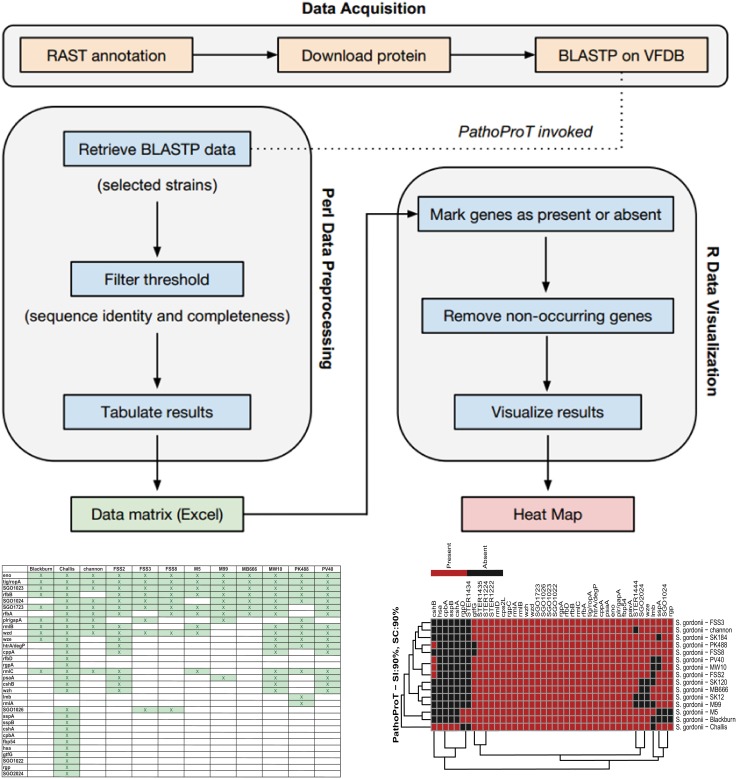
A PathoProT flowchart. PathoProT is mainly implemented using Perl and R scripts. The input of PathoProT would be lists of genes for the selected strains/genomes and the pipeline will generate a heat map at the end of the process.

Briefly, PathoProT pipeline was mainly implemented using Perl. In-house Perl scripts will process BLAST outputs (generated by searching these query sequences against VFDB) for each RAST-predicted protein (query sequence) in the user-selected genomes and identify putative virulence based on user-defined parameters. The filtered BLAST results are consolidated and organised in a matrix table containing information of presence or absence of virulence genes (rows) and *Streptococcus* strain names (columns). Finally, PathoProT will pass and process this output with our in-house R scripts for hierarchical clustering (complete-linkage algorithm) and generating a heat map for visualisation. The *Streptococcus* strains will be sorted based on their virulence gene profiles (Fig 4) and a phylogenetic tree will be drawn, users are able to gauge the relationships among the closely-related *S*. *mitis* group species/strains as well as their corresponding virulence genes form noticeable clusters through the dendrograms. Therefore, this comparative pathogenomics analysis pipeline is able to provide excellent insight into the virulence gene profiles across different species of *Streptococcus*. For instance, there is no existing bioinformatics tool that serves the same functionality as PathoProT, which is to predict and allow comparison of virulence genes across different species of bacterial genomes.

**Fig 5:**
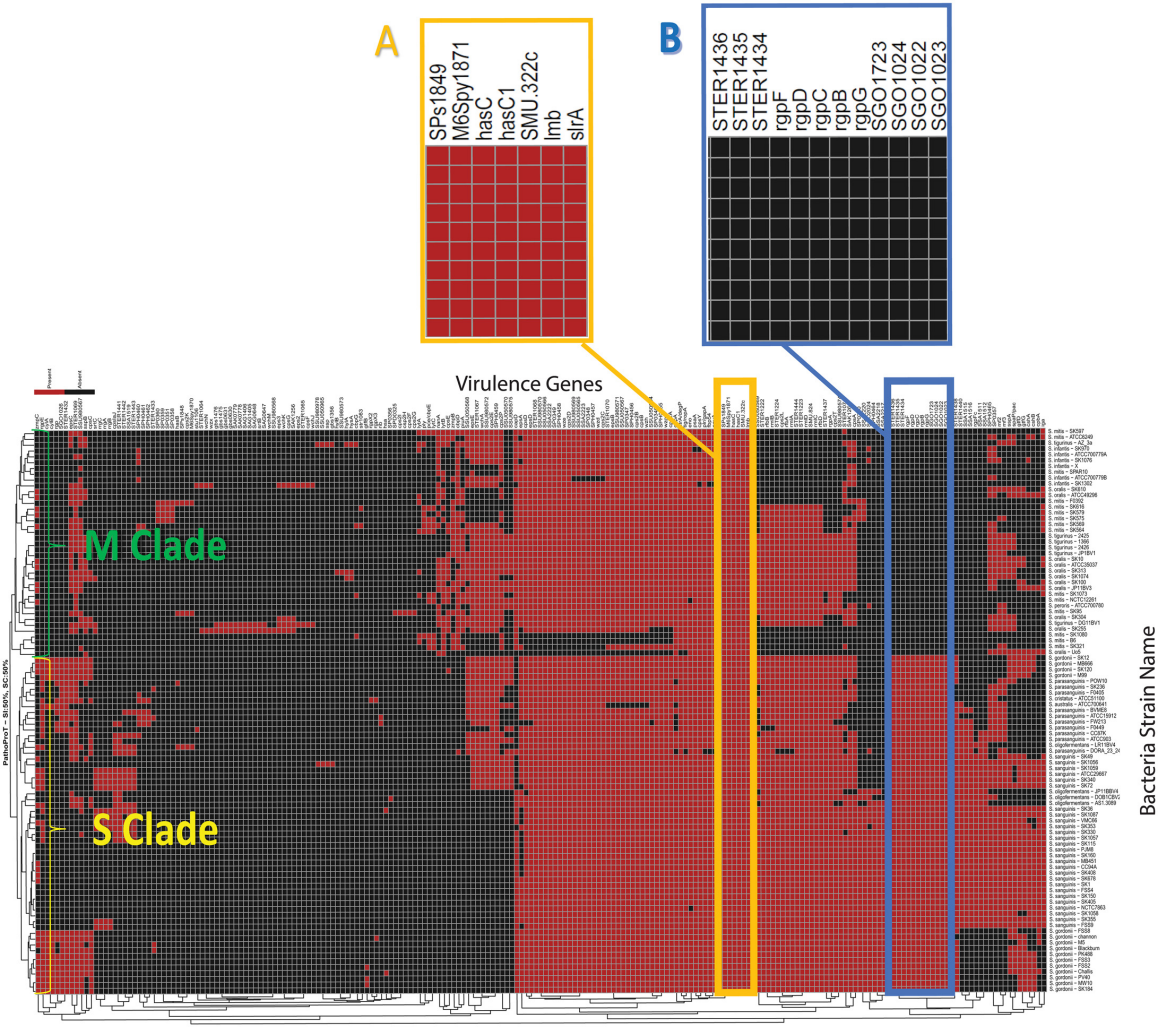
An informative heat map generated by PathoProT tool. (A) A list of conserved virulence genes carried by all mitis group species and (B) The RGP synthesis related genes which can differentiate M Clade and S Clade. Presence of the virulence gene was labeled in red and absence of the virulence genes was labelled in black.

To demonstrate the features or functionalities of PathoProT, we present a comparative pathogenomics study among the *S*. *mitis* group bacteria using a threshold of 50% for both sequence identity and coverage to give an insight into their virulence gene profiles. Based on the generated PathoProT heat map, a number of putative virulence genes appear to be conserved among all the mitis group species (Fig 5). The conserved genes *hasC (hasC1*or*SMU*.*322c)* which encodes UTP-glucose-1-phosphate uridylyltransferase (or UDP—glucose pyrophosphorylase)(M6Spy1871) is involved in synthesis of the hyaluronic acid (HA) capsule along with two neighboring genes: *hasA* and *hasB* within the *has* operon.[21]. In fact, *Streptococcus pneumoniae*, the most pathogenic species of the *S*. *mitis* group possesses a polysaccharide capsule which contributes to bacterial pathogenesis [22]. In *Streptococcus*, HA is found as streptococcal capsule material in some species is an important virulence factor, camouflaging the bacteria efficiently against the recognition of host immune system [23,24] as well as protecting them against reactive oxides released by leukocytes [25]. Additionally, it is possible that HA plays a significant role in mitis group streptococcal adherence and colonization of epithelial cells, leading to bacterial resistance against phagocytosis by macrophages [26–28].

Another conserved virulence gene, *slrA* encodes streptococcal lipoprotein rotamase A, which is one of the major surface proteins expressed by *S*. *pneumoniae*. This gene is an important cyclophilin which modulates biological function of virulence proteins during the first stage of pneumococcal infection [29]. It is likely that the *slrA* gene promotes invasion of host cells and facilitates pneumococcal colonization and adherence in *S*. *mitis* group bacteria[30,31]. Furthermore, it has been reported that deficiency in *slrA* reduces bacterial virulence due to its impact on the adherence and internalization by epithelial and endothelial cells [29]. Likewise, the conserved *lmb* gene encodes a laminin-binding protein which was first identified in *Streptococcus agalactiae* [32]. The virtually identical adhesins were later discovered in both *Streptococcus suis* [33] and *Streptococcus pyogenes* [34,35]. The *lmb* adhesins have been proposed to help in bacterial pathogenesis via invasion of the damaged epithelium [36]. Overall many surface lipoproteins and adhesins that are important in virulence and pathogenic infections are highly conserved across the *S*. *mitis* group bacteria.

According to the phylogenetics tree generated on the left side of the PathoProT heat map (Fig 5), the mitis group can be clearly categorized into two clades: S Clade (*S*. *sanguinis*, *S*. *gordonii*, *S*. *parasanguinis*, *S*. *australis*, *S*. *cristatus* and *S*. *oligofermentans*) and M Clade (*S*. *mitis*, *S*. *infantis*, *S*. *tigurinus*, *S*. *oralis* and *S*. *peroris*). This phylogeny relationship of the *S*. *mitis* group species indicates the close relatedness of cross-species within M Clade and species-to-species of S Clade. Interestingly, we found the *rgp* genes can be used to differentiate the two different clades in the heat map. For instance, these marker genes are present in all S Clade species but absent in all the M Clade species.

The *rgp* genes cluster (B, C, D, F and G) is responsible for the synthesis of rhamnose-glucose polysaccharide (RGP) in *Streptococcus mutans*. Notably, similar genes have been found to be involved in rhamnan synthesis in *Escherichia coli* [37]. In fact, it has been suggested that *E*. *coli* and *S*. *mutans* share a common pathway for rhamnan synthesis based on their similarities in RGP synthesis [37]. The function of *rgpB* is to transfer the second rhamnose residue to a rhamnose residue on ***N***-acetylglucosamine linked to the lipid carrier, followed by *rgpF* which later catalyzes the transfer of the third rhamnose residue to the second rhamnose residue of the resultant glycolipid carrier. Both *rgpB* and *rgpF* have presumably to work alternately in the elongation of the rhamnan chain. Homologous rhamnosyl transferases of *rgpB* and *rgpF* have been detected in *Streptococcus thermophilus* (STER1436) and *Streptococcus gordonii* (SGO1022). On the other hand, *rgpC* and *rgpD* genes encode the putative ABC transporters specific for RGP (homologous STER1434 in *S*. *thermophilus* and homologous SGO1024 in *S*. *gordonii*) which play role in polysaccharide export [37]. The *rgpG* gene (*S*. *gordonii* SGO1723 homolog) initiates the RGP synthesis by transferring *N*-acetylglucosamine-1-phosphate to a lipid carrier [38].

The *rgp* genes are also implicated in pathogenesis in several *Streptococcus* species. For instance, *rgp* plays an essential role in bacterial virulence as well as eliciting an inflammatory response in *S*. *suis* [39]. Induction of infective endocarditis by *S*. *mutans* has been reported to be triggered by *rgp* genes via nitric oxide release [40], platelet aggregation [41] and conferring resistance to phagocytosis by human polymorphonuclear leukocytes [42]. Therefore, S Clade *S*. *mitis* group species which produce these rhamnose rich polymers might exhibit a different pattern of pathogenesis from M Clade *Streptococcus* species in order to establish greater virulence and increased survival in host cells. A recent study has identified the Sanguinis group of streptococci as a common causative agent of transient bacteremia which potentially can lead to infective endocarditis. This group has also been reported to be present in a few cases of virulent septicemic infection in neutropenic patients [43].

#### Sequence search tools

We have incorporated two types of BLAST engines, standard BLAST and VFDB BLAST, into StreptoBase to search for the closest *Streptococcus* strains to the query strain. These exclusive BLAST searches are functionally based on the stand-alone BLAST tool [44] downloaded from NCBI. Both BLAST engines support three types of BLAST functions, namely, BLASTN, BLASTP and BLASTX. Users are allowed to define the genome completeness (%) and genome identity (%) on the BLAST tools submission forms. These specialized BLAST tools are aimed to facilitate users to perform similarity searches of their query sequences against *Streptococcus* genome sequences, gene sequences (standard BLAST) as well as against the virulence genes of VFDB (VFDB BLAST), which allows users to examine whether their genes of interest are potential virulence genes using a sequence homology approach.

### Future work and conclusion

With advances in NGS technology, further *Streptococcus* species or strains will be sequenced and this creates an urgent need to store, browse, retrieve and analyze vast amounts of genome data and the development of specialized tools for comparative analyses of these genomes.

Here we have successfully described and demonstrated the functionalities of StreptoBase particularly our in-house designed bioinformatics pipelines for the analyses of *Streptococcus* genomic data.

This specialized biological database will be constantly updated in order to provide the latest genome updates and research developments associated with the *Streptococcus* genus, and to ensure the accuracy and usefulness of the *S*. *mitis* group species genome data and annotation. We anticipate that StreptoBase will serve as a useful resource and analysis platform particularly for comparative analyses of the *S*. *mitis* group genomes for research communities. We encourage other researchers or research groups to offer suggestions and share their annotations, opinions, and curated data with us at girg@um.edu.my.

### Availability and system requirements

StreptoBase is available online at http://Streptococcus.um.edu.my. Users can download and visualize all sequences and annotations described in this paper on the StreptoBase website. Strains that have not already been deposited in the NCTC or ATCC culture collections are available on request from NSJ. This analysis platform is generally compatible with multiple type of browsers including Internet Explorer 8.x or higher, Mozilla Firefox^®^ 10.x or higher, Safari 5.1 or higher, Chrome 18 or higher and any other equivalent browser software. This web site is best viewed at a screen resolution of 1024 × 768 pixels or higher.

## Supporting Information

S1 Fig The genome overview of 104 *Streptococcus mitis* group genomes in StreptoBase. The genome details include genome size, number of contigs, number of ORFs, number of tRNAs, number of rRNAs, GC content as well as NCBI accession numbers of the 104 *Streptococcus* strains.
(XLSX)
